# Can brain natriuretic peptide predict cardiovascular complications in severe preeclampsia? A case-control study

**DOI:** 10.18502/ijrm.v17i4.4552

**Published:** 2019-06-13

**Authors:** Nayereh Ghomian, Farveh Vakilian, Bahram Shahri, Vesam Rostaminejad, Majid Khadem-Rezaiyan

**Affiliations:** ^1^Department of Obstetrics and Gynecology, Faculty of Medicine, Mashhad University of Medical Sciences, Mashhad, Iran.; ^2^Department of Cardiology, Faculty of Medicine, Mashhad University of Medical Sciences, Mashhad, Iran.; ^3^Department of Social Medicine, Faculty of Medicine, Mashhad University of Medical Sciences, Mashhad, Iran.

**Keywords:** *Brain natriuretic peptide*, * Echocardiography*, * Stroke volume*, * Pre-eclampsia, Pregnancy*

## Abstract

**Background:**

Preeclampsia is one of the most common pregnancy complications, which is one of the major causes of fetal and maternal mortality.

**Objective:**

To compare the serum Brain Natriuretic Peptide (BNP) level in patients with severe preeclampsia and normal pregnancy and investigate associated cardiovascular complications.

**Materials and Methods:**

This case-control study was performed on 94 women with a singleton pregnancy (severe preeclampsia and normal pregnancy) at Imam Reza Hospital in Mashhad, Iran. The venous blood samples were collected to evaluate the serum BNP level. All patients were subjected to echocardiography performed by a single cardiologist.

**Results:**

The mean pro-BNP levels were 480.36 ± 754.52 and 67.46 ± 106.24 pg/dL in the severe preeclampsia and normal pregnancy patients, respectively (p < 0.001). However, adjusted BNP for maternal and gestational age was not different in the two groups (ANCOVA, p = 0.69). Furthermore, the two study groups showed no significant difference in terms of the cardiac parameters, including ejection fraction (EF), left ventricle end-diastolic and -systolic diameters (LVEDD and LVESD, respectively), tricuspid annular plane systolic excursion (TAPSE), and *ratio* of the early transmitral flow velocity to the early diastolic tissue velocity (*E*/*Em*). However, the serum BNP level showed a significant correlation with EF (r = -0.39, p = 0.008), TAPSE (r = -0.47, p = 0.001), and E/Em ratio (r = 0.48, p = 0.001) in patients with severe preeclampsia.

**Conclusion:**

It seems that BNP can be used as a predictor for some of the main cardiac functional indices (i.e., E/Em, EF, and TAPSE) in severe preeclampsia patients.

## 1. Introduction

Gestational hypertension and preeclampsia are among the most prevalent medical problems during pregnancy, which mostly occur in the young and nulliparous females (1, 2). Preeclampsia is one of the main risk factors for maternal mortality along with maternal and fetal-neonatal complications (3). This condition complicates 5–8% of the pregnancies and has an unknown etiology (4). Preeclampsia occurs early in pregnancy through hidden pathophysiological changes that accelerate during pregnancy and ultimately becomes clinically apparent (2). Several investigations have been conducted to find the predictive factors of preeclampsia. However, there is no reliable, valid, and cost-effective method for predicting this condition. Preeclampsia can be diagnosed in its early stages only through the implementation of planned prenatal care (5). The first step in the prevention of preeclampsia is the early detection of women at risk of this complication. Therefore, the use of an appropriate diagnostic test that can help identify the patients at risk is of paramount importance. Such a test can play a significant role in the reduction of maternal and fetal mortality and morbidity (6). Severe cardiovascular dysfunctions are common in preeclampsia syndrome. These disorders increase the cardiac output due to high blood pressure and induce a significant negative impact on the cardiac input by the pathologic reduction of hypovolemia during pregnancy. The echocardiography of myocardial function and the clinical assessment of ventricular function should be considered when evaluating cardiac function in preeclampsia (7). Brain natriuretic peptide (BNP) is a polypeptide, which is secreted as a pre-pro BNP by the cardiac ventricle in response to *excessive stretching* of the *heart muscle cells *in the ventricular myocarditis and slightly stored in the granules (8–10). It has been recently demonstrated that the patients with preeclampsia have an elevated BNP level as compared to those with normal pregnancy, which is indicative of subclinical vascular disorders in these patients (11). Furthermore, the previous studies have demonstrated a direct relationship between the BNP level and the severity of preeclampsia and obstetrical complications, such as preterm delivery (12). According to various studies, cardiovascular disorders, such as increased systemic vascular resistance and left ventricular pressure, decreased heart rate, left ventricular diastolic dysfunction, and increased left ventricular mass index are associated with the elevation of BNP in preeclampsia. However, these studies have included both severe and non-severe preeclampsia (12).

With this background in mind, the present study aimed to compare the serum BNP level in patients with severe preeclampsia and normal pregnancy and investigating cardiovascular complications. The findings of this study may be helpful in introducing BNP as a marker for the identification of patients requiring cardiac evaluation.

## 2. Materials and Methods

This case-control study was performed on 94 women with singleton pregnancy at Imam Reza Hospital in Mashhad, Iran from September 2015 to November 2016. The study population who were selected among referral patients were patients with preeclampsia and those with normal pregnancy. The sample size of this study was estimated as 45 patients in each group based on a study conducted by Hamad and colleagues (13) and the formula for comparing the two means with an alpha of 5%, power of 80%, and 10% for drop out. The inclusion criteria were women with severe preeclampsia which is defined as the presence of one of the following signs or symptoms in the presence of preeclampsia: (i) a blood pressure of ≥ 160/110 mmHg after 20 wk of pregnancy; (ii) platelet count of less than 100,000 per microliter; (iii) an increase in liver enzymes twice normal; (iv) elevation of creatinine higher than 1.1 mg/dl or doubling of baseline; and (v) headache, blurred vision, and epigastric pain. Eclampsia itself was diagnosed based on the American College of Obstetricians and Gynecologists' Guideline (14). On the other hand, the exclusion criteria were: (i) known chronic diseases including cardiac, pulmonary, renal, connective tissue disorders, hepatic diseases, and hyperthyroidism; (ii) body mass index of > 30 or < 18.5; (iii) diabetes; (iv) history of peripartum cardiomyopathy, heart valve disease, and severe electrocardiographic abnormalities; (v) consumption of cardiac medication; and (vi) chronic hypertension. The control group was selected from the mothers with normal pregnancy (i.e., with no history of any disease in these women and did not develop any complications during pregnancy or labor).

A checklist containing maternal age, gravidity, gestational age, systolic and diastolic blood pressure, number of previous pregnancies, body mass index, and drug consumption was filled. To evaluate the serum BNP level, 2–4 cc venous blood samples were collected from the preeclampsia (before the administration of magnesium sulfate) and control groups in the biochemistry tube (gelled) stored at 4°C in the refrigerator and sent to the laboratory. After being exposed to ambient temperature for 30 min, pro-BNP was measured using VIDASⓇ NT-proBNP2 kit (Biomerieux, France) and advanced enzyme-linked immunosorbent assay. In addition, to determine the electrocardiographic variations, all patients were subjected to a standard b-mode transthoracic echocardiography (Samsung V10 model) performed by a single cardiologist who was blind to the patient's status regarding blood pressure. The echocardiography was conducted before the administration of magnesium sulfate.

### Ethical consideration

This study was approved by the Research Chancellor of Mashhad University of Medical Sciences, Mashhad, Iran (940284). Ethical Committee of Mashhad University of Medical Sciences approved the ethical issues regarding the involvement of human subjects (IR.MUMS.fm.REC.1394.355). Informed consent was obtained from all participants.

### Statistical analysis

Data analyses were performed using Statistical Package for the Social Sciences, version 16.0, SPSS Inc, Chicago, Illinois, USA (SPSS). The relationship between the qualitative and quantitative variables was evaluated using the student *t*-test, and Mann-Whitney U test. ANCOVA was used to control confounders. Furthermore, the correlation between the quantitative variables was estimated through Spearman's rank correlation coefficient. All tests were two-tailed and P-value less than 0.05 was considered statistically significant.

## 3. Results

Overall, 94 pregnant women (i.e., 44 participants with severe preeclampsia and 50 participants with a normal pregnancy) were studied. Except for age and gestational age, there was no significant difference between the normal and preeclampsia groups (Table I). The mean pro-BNP levels in the women with preeclampsia (480.36 ± 754.52 pg/dl) were significantly higher than women with normal pregnancy (67.46 ± 106.24 pg/dl, Mann-Whitney Test, p < 0.001). However, after controlling for age and gestational age, this difference was diminished (ANCOVA, p = 0.69).

**Figure 1:**
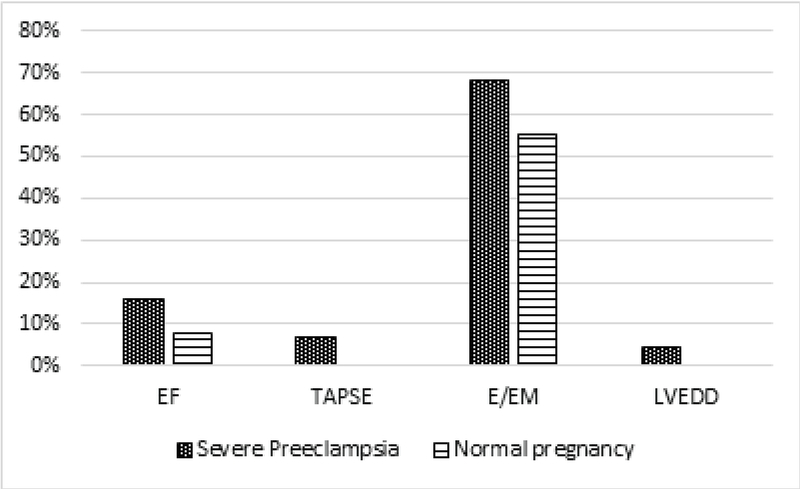
Comparison of abnormal echocardiographic indices (ejection fraction (EF), tricuspid annular plane systolic excursion (TAPSE), E/Em ratio, left ventricular end-diastolic diameter (LVEDD)) in the two study groups.

**Figure 2:**
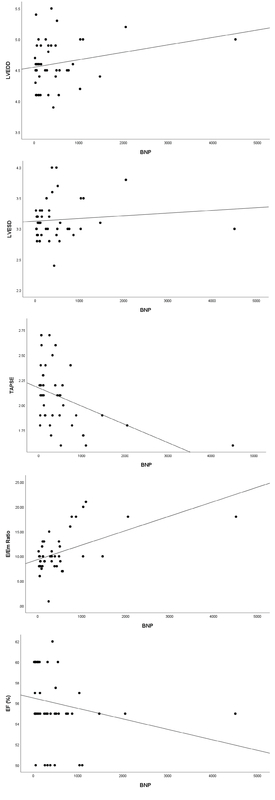
Scatter plot for the correlation of ejection fraction (EF), tricuspid annular plane systolic excursion (TAPSE), E/Em ratio, left ventricular end-diastolic diameter (LVEDD), and left ventricular end-systolic diameter (LVESD) with blood natriuretic peptide (BNP) level in patients with severe preeclampsia.

There was no significant difference between the two groups (neither before or after controlling for age and gestational age as confounders) in terms of the cardiac parameters, including EF, LVEDD and LVESD, TAPSE which is an indicator of the right ventricular systolic function, and *ratio* of the early transmitral flow velocity to the early diastolic tissue velocity (*E*/*Em) which is *an index for the left ventricular diastolic function (Table II). Considering EF ≥ 55%, LVEDD < 5.3, TAPSE ≥ 1.7, and E/Em ≤ 8 as normal values, and categorizing quantitative variables into dichotomous ones, it was revealed that although all echocardiographic indices were more frequently abnormal in women with severe preeclampsia than women with normal pregnancy, no statistically significant difference was found (Figure 1). The serum BNP level had a negative correlation with EF (r = -0.39, p = 0.008), TAPSE (r = -0.47, p = 0.001) and a positive correlation with E/Em ratio (r = 0.48, p = 0.001), LVEDD (r = 0.15, p = 0.30), and LVESD (r = 0.11, p = 0.47) in patients with severe preeclampsia (Figure 2).

**Table 1:** Comparison of the demographic variables between the patients with severe preeclampsia and normal pregnancy


	**Normal pregnancy (n = 50)**	**Severe preeclampsia (n = 44)**	**p-Value**
Age (yr)	26.46 ± 6.00	30.86 ± 7.01	0.001
Gestational age (wk)	38.60 ± 1.86	33.60 ± 4.64	0.001
Body mass index (kg/m${}^{2}$)	29.69 ± 6.27	27.32 ± 5.33	0.55
Parity (n)	2.34 ± 1.17	2.63 ± 1.61	0.33
white<bcol>4</ecol>Note: All data are reported as mean ± SD; Student *t*-test was used for comparison

**Table 2:** Comparison of echocardiographic indices in the study groups


	**Normal pregnancy (n = 50)**	**Severe preeclampsia (n = 44)**	**p-value**
Ejection fraction (%)	57 (55–60)	55 (55–60)	0.25
Left ventricular end-systolic diameter (cm)	3.1 (2.9–3.2)	3.1 (2.9–3.3)	0.28
Left ventricular end-diastolic diameter (cm)	4.7 (4.4–4.9)	4.5 (4.4–4.9)	0.94
Tricuspid Annular Plane Systolic Excursion (cm)	2.2 (1.9–2.5)	2.1 (1.9–2.2)	0.07
E/Em(ratio)	9 (7–11)	10 (8.0–12.7)	0.06
white<bcol>4</ecol>Note: All data are reported as Median (IQR); Mann-Whitney test was used for comparison

## 4. Discussion

The aim of this study was to compare serum BNP levels in the patients with severe preeclampsia and normal pregnancy group. We also determined the relationship of serum BNP level with the left ventricular systolic and diastolic dysfunction in the patients with severe preeclampsia. In the present study, the mean total pro-BNP level showed an increase in patients with severe preeclampsia in comparison to the normal pregnancy group. However, after adjusting for maternal and gestational age statistical differences was not seen. Additionally, some echocardiographic indices, such as E/Em ratio, LVEDD, and LVESD, had a positive correlation with BNP in patients with severe preeclampsia. These findings suggested that BNP can be used as a marker for the diagnosis of cardiac complications. On the other hand, EF and TAPSE had a negative correlation with BNP in patients with severe preeclampsia. It should be noted that the mean NT-pro BNP level obtained in the present study was higher than those reported in previous studies (13, 15). However, there was no significant difference between the two groups in terms of underlying heart disease and renal function. The possible reason of obtaining a high mean BNP in the present study in comparison to those obtained in the other studies could be ascribed to high levels of BNP in two patients with preeclampsia who had left ventricular dysfunction and a significant reduction in EF ratio. These two patients had no known underlying heart disease, and we could not find any etiology for their condition during the investigation. Therefore, they were not excluded from the study, and it was assumed that high BNP and cardiac findings of these two patients might be affected by their preeclampsia severity.

In a study conducted by Resnik and colleagues in 2005, the BNP levels of 25 healthy pregnant women with 34 preeclamptic women were compared. In the mentioned study, the median levels of BNP in the normal subjects and the patients with moderate and severe preeclampsia were 17.8, 21.1, and 101 pg/ml, respectively. "This finding indicated the significantly higher level of this hormone in the severe preeclampsia group than that in the mild preeclampsia group, which could reflect the ventricular stress or subclinical cardiac disorder associated with preeclampsia" (15).

There are different studies examining the association of BNP with the severity of preeclampsia. For instance, Bakacak and colleagues conducted a study on 49 females with preeclampsia, 25 of whom had severe preeclampsia, and 27 pregnant women with normal blood pressure (16). "Their findings showed that serum NT-pro BNP level in the preeclampsia group was significantly higher than that in the control group. In addition, the NT-pro BNP level was significantly higher in the severe preeclampsia group than those in non-severe preeclampsia and control groups". Therefore, the mentioned study concluded the NT-pro BNP level as a useful marker for estimating the severity of preeclampsia (16).

Hamad and colleagues also performed a study in 2009 on 35 pregnant women with preeclampsia and 30 females with normal pregnancy. They performed echocardiography and estimated the NT-pro-BNP, C-reactive protein, cystatin C, and troponin I level during pregnancy and 3–6 months after the delivery. The results demonstrated that the function and dimensions of the left ventricle and atria were significantly different between the two groups. In addition, they reported a significant increase in the septal and lateral E/Em ratio in the preeclampsia group. Also, the levels of NT-pro-BNP and cystatin C were demonstrated to significantly elevate in the preeclampsia group (13). However, we did not find a statistically significant difference between the two groups regarding cardiac indices which could be due to the period of exposure with preeclampsia (i.e., from diagnosis to cessation of pregnancy. Also, in previous reports, patients with early onset preeclampsia were studied but in our study, patients with severe eclampsia at all gestational ages were included.

In another study conducted by Fayers and colleagues in 2013, the BNP levels and echocardiographic variations of 52 patients with preeclampsia and 63 healthy pregnant women were investigated (17). The results were indicative of the elevated level of BNP in the females with preeclampsia along with changes in the left ventricular diastolic function as compared with the females with normal pregnancy. Our results were similar to those reported by Fayers and colleagues (17) in terms of the enhancement of pro-BNP levels in the preeclampsia group. Nonetheless, the examination of cardiac parameters based on echocardiography revealed that the two groups of preeclampsia and normal pregnancy showed no significant difference in this regard. However, in the severe preeclampsia group, BNP levels had a significant correlation with EF, TAPSE, and E/Em. This could be indicative of the right and left ventricular systolic and diastolic dysfunction.

In a systematic review conducted on 12 studies in 2013, the BNP level was reported to be significantly higher in the preeclampsia patients than that in normal pregnancies. Additionally, in the mentioned study, the high level of BNP was reported to be associated with cardiovascular complications and preterm delivery. However, the researchers of the mentioned study suggested performing more studies on the patients with preeclampsia in order to obtain more accurate findings in this regard (1).

It is noteworthy that although there are some studies investigating BNP and preeclampsia, limited research has examined the relationship between cardiac parameters in preeclampsia and BNP levels. To the best of our knowledge, this was the first study to evaluate the correlation of BNP with cardiac indices in preeclampsia in the east of Iran. We also suggest to include pregnancy complications and neonatal outcome in future studies.

## 5. Conclusion

In conclusion, the total mean Pro-BNP level was not statistically different in patients with severe preeclampsia in comparison to that in the normal pregnancy. However, E/Em had a statistically positive correlation with BNP and EF and TAPSE had a negative correlation with BNP in severe preeclampsia patients. So, BNP can be used as a predictor for at least some of the main cardiac functional indices in patients with severe preeclampsia.

##  Conflict of Interest

The authors declare that they have no conflict of interest.
